# Contrasting Effects of Historical Sea Level Rise and Contemporary Ocean Currents on Regional Gene Flow of *Rhizophora racemosa* in Eastern Atlantic Mangroves

**DOI:** 10.1371/journal.pone.0150950

**Published:** 2016-03-10

**Authors:** Magdalene N. Ngeve, Tom Van der Stocken, Dimitris Menemenlis, Nico Koedam, Ludwig Triest

**Affiliations:** 1 Laboratory of Plant Biology and Nature Management (APNA), Department of Biology, Vrije Universiteit Brussel (VUB), Pleinlaan 2, B-1050, Brussels, Belgium; 2 Earth Sciences Division, Jet Propulsion Laboratory, California Institute of Technology, Pasadena, California, United States of America; Griffith University, AUSTRALIA

## Abstract

Mangroves are seafaring taxa through their hydrochorous propagules that have the potential to disperse over long distances. Therefore, investigating their patterns of gene flow provides insights on the processes involved in the spatial genetic structuring of populations. The coastline of Cameroon has a particular geomorphological history and coastal hydrology with complex contemporary patterns of ocean currents, which we hypothesize to have effects on the spatial configuration and composition of present-day mangroves within its spans. A total of 982 trees were sampled from 33 transects (11 sites) in 4 estuaries. Using 11 polymorphic SSR markers, we investigated genetic diversity and structure of *Rhizophora racemosa*, a widespread species in the region. Genetic diversity was low to moderate and genetic differentiation between nearly all population pairs was significant. Bayesian clustering analysis, PCoA, estimates of contemporary migration rates and identification of barriers to gene flow were used and complemented with estimated dispersal trajectories of hourly released virtual propagules, using high-resolution surface current from a mesoscale and tide-resolving ocean simulation. These indicate that the Cameroon Volcanic Line (CVL) is not a present-day barrier to gene flow. Rather, the Inter-Bioko-Cameroon (IBC) corridor, formed due to sea level rise, allows for connectivity between two mangrove areas that were isolated during glacial times by the CVL. Genetic data and numerical ocean simulations indicated that an oceanic convergence zone near the Cameroon Estuary complex (CEC) presents a strong barrier to gene flow, resulting in genetic discontinuities between the mangrove areas on either side. This convergence did not result in higher genetic diversity at the CEC as we had hypothesized. In conclusion, the genetic structure of *Rhizophora racemosa* is maintained by the contrasting effects of the contemporary oceanic convergence and historical climate change-induced sea level rise.

## Introduction

The potential effects of climate change on coastal ecosystems have received heightened attention recently [1]. Mangrove ecosystems are considered resilient under changing environmental conditions [2–5], with potential expansion in some areas [1, 5]. Landward expansion, for example, has been reported in localities where overall accretion keeps pace with relative sea level rise (SLR) *i*.*e*., accretionary surplus [1, 2, 6, 7], while pole-ward (latitudinal) expansion may occur in response to rising temperatures, *i*.*e*., higher average winter temperatures [2, 8–10]. SLR may also favor landward mangrove expansion by decreasing subsidence, increasing saline intrusion, and changing the ground water level in coastal areas, thereby allowing mangroves to thrive over other vegetation [1]. Besides SLR, changing climatic conditions also include changes in the frequency and intensity of extreme meteorological events such as tropical cyclones [11]. Although solid evidence is yet missing, such meteorological trends may favor biogeographic range expansion through fostering long distance dispersal (LDD) by increasing the vector (ocean surface current) seed load, vector displacement velocity, and seed passage time [12]. Dispersal processes over oceans have shaped ranges of mangroves and the genetic structure of populations throughout the species’ existence, but evidence is needed as to the timescale and settings which define the outcome that are actually observed. Understanding dispersal processes of the past may give insight to species responses to future climate change.

Mangrove ecosystems occur along coastal areas in the (sub)-tropics, approximately between 30° N and 30° S. Their socio-economic and ecological importance has been widely recognized [13–18], rendering them an integral part of the daily lives of many coastal communities. The importance of these ecosystems, however, is not restricted to their biogeographical extent. Despite occupying only 0.7% of the tropical forest area, mangrove degradation may contribute to more than 10% of global carbon emissions due to deforestation [19]. Nevertheless, global mangrove stocks have decreased more than 40% over the last 50 years, with a continuing yearly decline of 2% [20]. By the year 2000, global mangrove cover was estimated to be 137760 km^2^ [21]. Fossil evidence indicates that mangroves once occupied a broader geographical range than they do today [22–24] due to spatial changes in favorable temperatures. It has been hypothesized that cold temperature, in interaction with aridity, are the delimiting factors to mangrove range expansions [25–27]. Climatic oscillations of the past have also been characterized by successive range expansions and contractions for many mangrove species; range expansions after the Last Glacial Maximum (LGM) have been reported for many Neotropical species [25, 27–29]. The present distribution of mangroves is shaped by (1) suitable intertidal habitats, (2) the potential of propagules to reach these locations within their viable periods [30, 31], and (3) various thresholds to establishment [6]. The hydrochorous propagules of *Rhizophora* can remain viable and afloat for more than 3 months [32], providing a sufficiently long time window for potential LDD and the colonization of new favorable habitats. Nevertheless, multiple barriers may hamper a species from colonizing its full potential niche (*sensu* Randin *et al*. [33]), for example unfavorable ocean currents, closed estuaries, arid [34] or hard rock coastlines, and the physical disturbance by tidal inundation and wave-induced sediment dynamics [31, 35].

These factors, in addition to local pollen flow, determine the overall genetic structure as they control the exchange and fluxes of alleles between nearby and remote populations. Absence of or limited gene flow can result from vicariance [36–38], as well as distance-related dispersal limitation, where genetic differentiation shows a linear relationship with geographical distance between populations [39]. Orsini *et al*. [40] postulated that isolation of natural populations could indeed be due to either (1) distance-related dispersal limitation (Isolation by Distance; IBD), or (2) adaptation where selection pressure drives local adaptation, thereby reducing establishment success of non-adapted immigrants (Isolation by Adaptation; IBA). Also, (3) local adaptation combined with the numerical advantage (density-dependence) of first colonists (founder effect) may contribute to a strong priority effect of residents over immigrants, thereby creating isolation by colonization (IBC) [40, 41]. However, the source of colonizers, which could either follow a migrant pool model, where colonizers originate from a pool of different but still highly connected demes (thus resulting in low differentiation despite multiple sources), or a propagule pool model, where colonizers come from a pool of already differentiated origin population (thus resulting in high differentiation) [42], is also of great significance. The use of IBD models for explaining spatial genetic differentiation among populations, dominate literature [43], irrespective of the frequent observation of dissociation between genetic differentiation and geographical distance in marine systems [44] and the erroneous assumptions associated with the use of IBD models. IBD analysis generally assumes spatial homogeneity and/or theoretically unjustified distance metrics, although landscapes are naturally heterogeneous [45]. McRae [45] proposed the use of isolation by resistance model (IBR) which takes into account landscape heterogeneity. Information on barriers to gene flow, such as ocean currents, is used to define a resistance surface, predict spatial genetic structure of populations, and to explain deviations from the widely applied IBD model [44].

In mangroves, high levels of gene flow (Fst < 0.2, Nm > 1) along the same stretch of coastline have been reported by several authors [46–48], likely as a result of range expansion after the LGM (28). However, genetic structure at distance classes < 1000 km in the Atlantic East Pacific (AEP) suggests high levels of short distance dispersal (SDD) (and therefore low levels of long distance dispersal), at least for *Avicennia* spp. [28, 49]. Generally, broad-ranged seafaring species exist as patchy subpopulations separated by weak barriers [50]. Several barriers to gene flow among mangrove populations have been identified worldwide, resulting in patterns of genetic discontinuities. Land barriers like the Central American Isthmus, the Malay Peninsula and the Indonesian archipelago [37, 38, 51], as well as cold currents [28] and oceanic barriers, even at spatially proximate populations [28, 52, 53] have been well established. Despite the importance of such insight in explaining and understanding the genetic structure of mangrove populations across vast expanses of ocean or along coastlines at local, regional and continental scales, information on the underlying drivers that shape the genetic structure of mangroves along the East-Atlantic coast is missing.

In this study, we assess the genetic diversity and structure of *Rhizophora racemosa* G. Mey. populations from 4 estuaries along the coast of Cameroon. It is likely that the historical and current barriers, as well as the recent dispersal corridor on this coastline (see “Study area” below) may explain, at least partly, the configuration and composition of present-day mangrove forest in this area (*ca*. 400 km, which makes up *ca*. 6% of the mangrove coastline of the East Atlantic). The spatial configuration of mangroves along this coastline relative to past and present-day oceanographic and geomorphologic characteristics provides an interesting case to test the hypotheses that: (1) the historical barrier of the Cameroon Volcanic Line (CVL) (at the Bioko-mainland connection) has affected gene flow such that populations on either side of this barrier are isolated; (2) the presence of an ocean current convergence zone represents an important oceanic barrier to gene flow causing genetic discontinuity between populations on both sides of its frontal zone; and (3) the ocean current convergence zone results in higher genetic diversity of mangroves of the Cameroon Estuary complex (CEC) than among mangrove populations on either side of this convergence zone, due to input of propagules from areas north and south of it.

## Materials and Methods

### Ethics Statement

Permission to collect samples in the Campo Ma’an National Park and the Douala-Edea Reserve at Mouanko was obtained from the Ministry of Forestry and Wildlife (Ministère des forêts et de la faune). Local chiefs and local people gave permission to collect samples in non-protected areas. No specific permission was required for locations outside the protected areas and field studies did not involve endangered or protected species.

### Study area

**Fig 1:**
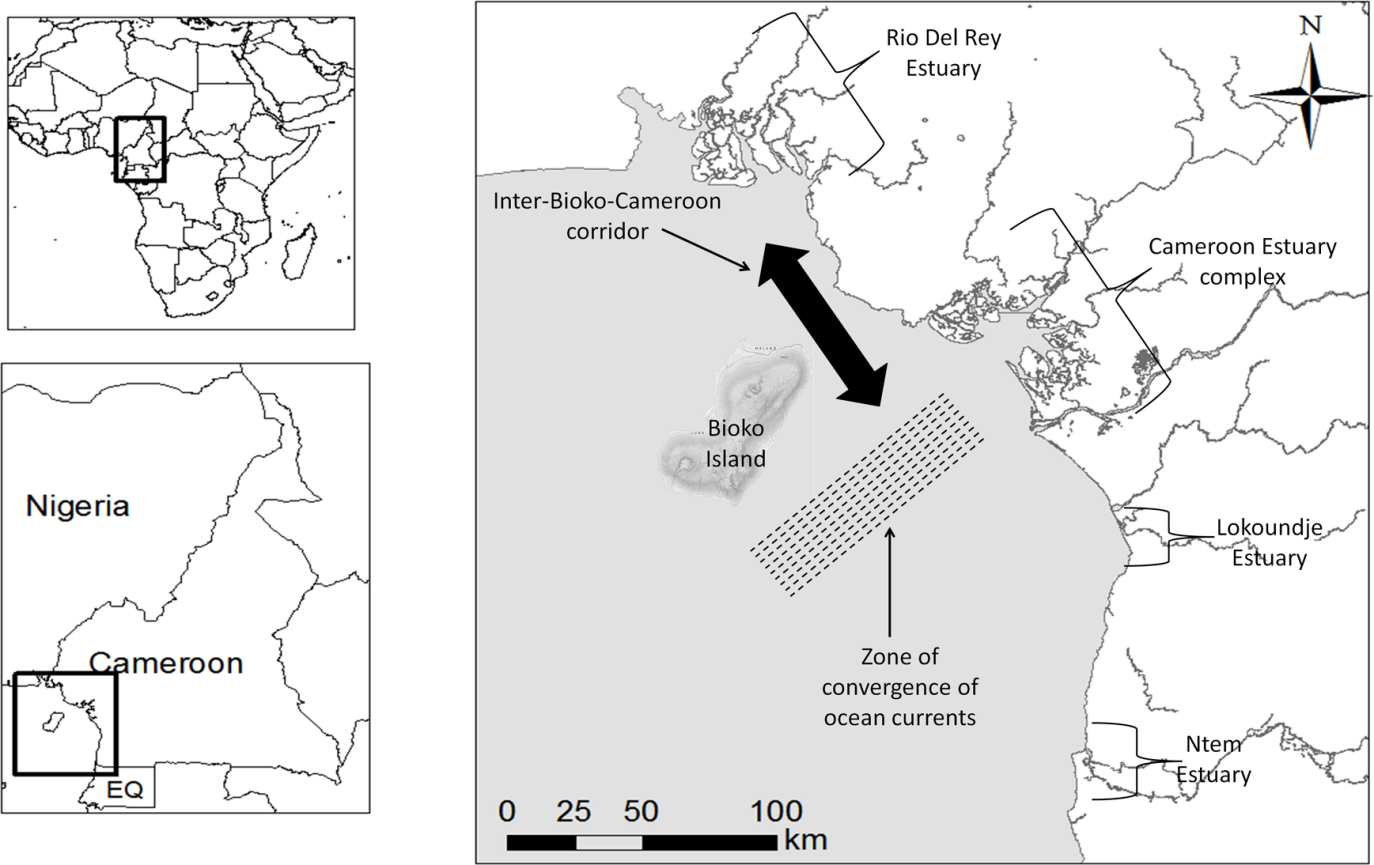
The coastal area of Cameroon indicating the studied estuaries and major dispersal corridor and barrier to gene flow. Map generated from Hydrography shape files obtained from the World Resource Institute (WRI) Congo Basin Forest Atlases webpage: http://www.wri.org/our-work/project/congo-basin-forests/cameroon.

The coast of Cameroon is about 400 km long, stretching from the country’s border with Equatorial Guinea in the south to its northern border with Nigeria, on the Eastern Atlantic coast. This coastline has an interesting geomorphological history and a coastal hydrology that is characterized by a dense network of rivers. The Cameroon Volcanic Line (CVL) is a 1600 to 1800 km chain of volcanoes [54, 55] that started forming about 30 Ma BP and stretches from far inland in the north, via Mount Cameroon and Mount Etinde across the Cameroonian Atlantic coast, to the Bioko, Príncipe, São Tomé, and Annobón Islands [56]. The CVL separates the mangroves of the present-day Rio Del Rey Estuary in the north from those of the Cameroon Estuary complex (CEC) and other southerly-situated populations (Fig 1). Bioko Island is the youngest of all these islands, having formed about 1 Ma BP, and it is situated *ca*. 40 km from the edge of the continental shelf at less than 60 m below sea level [56, 57]. It was repeatedly connected to mainland Cameroon during glacial times [56–58]. Following glacial retreat *ca*. 12 ka BP, sea level rose and covered these lowlands, separating Bioko from the mainland [56–58] and forming the Inter-Bioko-Cameroon (IBC) corridor. In addition to the many rivers flowing into the coastal waters of Cameroon, the isolation of Bioko Island from the mainland has caused ocean swells around the coast of Cameroon to be weak [59]. The tidal system in our study site is semidiurnal, ranging in amplitude between 0.3 m and 3 m, depending on the locality [59, 60]. Ocean currents near the coast of Cameroon are characterized by the convergence of the south-eastwards flowing Guinea Current and the northwards surface flow, converging near the CEC (Fig 1). Four disjunct mangrove areas (estuaries) currently exist along the coast of Cameroon with *Rhizophora racemosa* (90%) and *Avicennia germinans* L. (5%) being the predominant mangrove species on this coastline [61]. Hence, this study on genetic patterns of a widespread mangrove species, *R*. *racemosa*, reveals the importance of geomorphological history and ocean currents in shaping contemporary genetic structure.

### Sampling and genotyping

**Fig 2:**
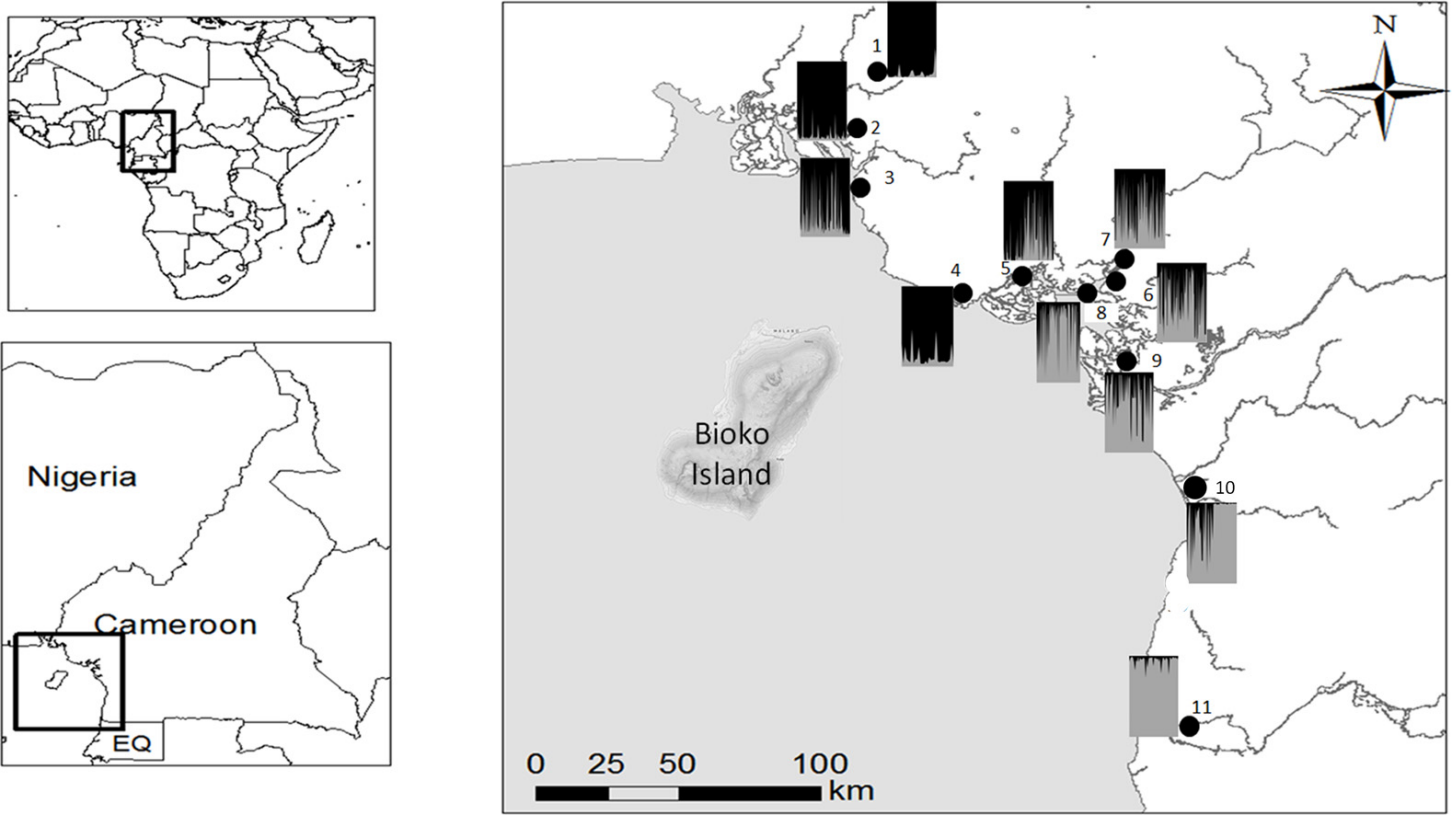
The coastal area of Cameroon with the 11 sites as in Table 1 and the proportions of two inferred clusters of individuals for each population. Proportions of inferred clusters (Q-values) per population are represented as bar charts at their respective geographical locations on the map.

Leaf tissue was collected from adult trees at least 10 m apart along transects (parallel to the direction of river flow), except at Mabeta where only fringing stands were left, hampering continuous transects. Hence, in the latter area, all adult individuals were sampled. On average, 30 adult trees were sampled per transect and transects in each site were pooled into a single population, except for areas where only one transect could be obtained due to very few stands (Ekondo-Titi and Mabeta). Three populations were sampled from the Rio Del Rey Estuary in the north (Ekondo-Titi, Mbongo, Bekumu), six populations from the CEC at the center (Mabeta, Tiko, Douala Akwa-Nord, Douala Bonamoussadi, Douala Bonaberi, Douala-Edea Reserve). The Kibi population from the Lokoundje Estuary (with transects from Londji and at Mpalla), and the Campo population from the Ntem Estuary (with transects from Ipono and Campo Beach) (see Table 1, Fig 1, Fig 2). Leaf samples were kept dry on silica gel. Genomic DNA was extracted from dried leaf tissue using the E.Z.N.A SP plant DNA Mini Kit (Omega bio-tek). Eleven (11) polymorphic microsatellite markers (Rrace1, Rrace3, Rrace5, Rrace6, Rrace7, Rrace12, Rrace15, Rrace17, Rrace18, Rrace20, and Rrace24) initially developed from Cameroonian populations of *R*. *racemosa* [62], were used in a single multiplex for the Polymerase Chain Reaction (PCR). The PCR reactants were 1.25 μl of the primer mix, 6.25 μl of the Master mix, 2.5 μl of H_2_O, and 3 μl of DNA, in a final volume of 13 μl. The PCR reaction conditions were: an initial denaturation of 95°C for 15 minutes followed by an extension of 30 seconds at the same temperature. Annealing was then allowed at a temperature of 57°C, followed by an extension at 72°C. Subsequently, the initial steps were repeated with final extension time of 60°C for 30 minutes and a cooling to 4°C for 1 minute in a Bio-Rad thermal cycler (MJ research PTC-200 and Bio-Rad MyCycler). Fragment analysis of PCR products was done by Macrogen Corporation (Seoul, South Korea). GeneMarker (SoftGenetics LLC, State College, USA) was used to score fragments.

**Table 1:** Description of the studied *R*. *racemosa* populations from the coastline of Cameroon.

Estuary	Site No.	Sites (Region)	Pop ID	Latitude	Longitude	Notes	Habitat type
**Rio Del Rey Estuary**	1	Ekondo-Titi	EKO	4°46'18.88'' N	8°47'52.84'' E	S, D,	Riverine
	2	Mbongo, Bekiri Beach	MBO	4°29'34.41'' N	8°55'51.00'' E	L,U	Riverine
	3	Bekumu	BEK	4°22'49.84'' N	8°53'46.45'' E	L, D	Estuarine
**Cameroon Estuary (CEC)**	4	Mabeta Njanga	MAB	3°57'53.09'' N	9°15'58.06'' E	S, D,	Coastal
	5	Tiko beach	TIKO	4°03'30.02'' N	9°22'56.06'' E	L, D	Riverine
	6	Douala, Akwa-Nord	AKN	4°05'23.76'' N	9°43'19.24'' E	S, D,	Riverine
	7	Douala, Bonamoussadi	SADI	4°06'51.60'' N	9°44'58.07'' E	S, D,	Riverine
	8	Douala, Bonaberi	BERI	4°04'40.70'' N	9°42'16.31'' E	S, D,	Estuarine
	9	Douala-Edea Reserve	RSVM	3°45'20.75" N	9°42' 0.26" E	L, U	Riverine
**Lokoundje**	10	Kribi, Londji & Mpalla	KRIBI	3°04'57.67'' N	9°58'29.50'' E	S, D,	Estuarine
**Ntem**	11	Campo Beach, Ipono	CAMPO	2°20'09.49'' N	9°51'19.89'' E	S, U,	Estuarine

S = small, L = Large, U = undisturbed, D = disturbed

### Data quality and genetic diversity

Genotypic linkage disequilibrium between all pairs of loci was tested using FSTAT v.1.2 [63]. The presence of null alleles and significant deviations from Hardy-Weinberg equilibrium (HWE) were investigated for each locus and population with Genepop v.4.3 [64]. MICRO-CHECKER [65] was used to investigate scoring errors and allele dropouts. We calculated allelic richness (Ar) and significance of heterozygote deficiency (Fis) based on Bonferroni corrections with FSTAT. Rarefaction of allelic richness was to the smallest population size (smallest sample = 30). The observed and expected heterozygosity were calculated using GenAlex v.6.5 [66]. The total number of alleles and number of effective alleles per population was obtained from GenAlex. We tested populations for recent bottleneck events with the software BOTTLENECK v.1.2.02 [67]. This was done with 1000 iterations by testing the assumptions that: mutation rate of microsatellite markers follow (1) an Infinite Allele Model (IAM); (2) a Stepwise Mutation Model (SMM); or (3) a Two Phase Model (TPM) (TPM assumes 70% SMM rate and a variance of 30%).

### Genetic structure

Global mean population differentiation (Ѳ) and inbreeding (f) over all loci [68] was estimated using FSTAT. Genetic differentiation, at first, was tested by a hierarchical analysis of molecular variance (AMOVA-Fst) calculated with GenAlex, where populations were grouped according to their estuary, and also by an AMOVA without any prior grouping into estuaries. Secondly, a Bayesian clustering analysis at individual level was conducted in STRUCTURE v.2.3.4 [69] following the admixture model by testing K values ranging from 1 to 10 without any prior indication of population origin, with 10 iterations per K value. The length of burn-in period was set at 10 000 and the number of Markov Chain Monte Carlo (MCMC) repeats after burning at 1 000 000. The program minimizes deviations from the Hardy Weinberg Equilibrium (HWE) and linkage equilibrium. The results of K values were obtained from STRUCTURE HARVESTER [70] and the Evanno method of the highest ΔK value [71] was used to determine the best K value. We also calculated pairwise genetic differentiation (Fst) with GenAlex and pairwise allelic differentiation (D_est_) using SMOGD online http://www.ngcrawford.com/django/jost/ [72], for all population pairs. Also patterns of the spatial relationship among populations was done by Principal Coordinate Analysis (PCoA) with GenAlex and by a Neighbor-joining (NJ) tree based on Nei’s genetic distances [73] as computed with POPTREE v.2 [74], bootstrapping at 10000.

### Hypotheses testing

#### The Cameroon Volcanic Line is a barrier to gene flow vs the Inter-Bioko-Cameroon corridor as a pathway for gene flow

It is expected that (1) if the CVL presents a barrier to gene flow, populations on either side of this barrier will be genetically isolated from each other and (2) if the Inter-Bioko-Cameroon corridor is a pathway for gene flow, populations will be admixed. To test this, we first created a predictor matrix whereby population pairs consisting of one population to the north and another to the south of this barrier were coded as 1 (high expected differentiation), while those with pairs located on the same side, either north or south, were coded as 0 (low expected differentiation), similar to the methodology applied by Dodd *et al*. [36] and Wee *et al*. [53]. This predictor matrix and a matrix of pairwise Fst were then used together in a Mantel test with 9999 permutations in GenAlex. Using BayesAss ed. 3 [75], we estimated contemporary migration (dispersal) rates and directionality between populations on either side of the supposed barrier or present-day corridor to check for connectivity between these areas. The program was run at 3 x 10^6^ MCMC iterations with a burn-in period of 10^6^ at a sampling frequency of 2000. This software allows only for the estimation of recent migration rates, *i*.*e* within the past 3 generations [38].

#### The convergence zone of two currents at the Cameroon Estuary complex is a barrier to gene flow

We expect that if the zone of convergence of the Guinea Current and the northward flowing surface current is a barrier to gene flow, there will be a genetic discontinuity and an absence of migration between populations on either side of the barrier. Contemporary migration (dispersal) rates were calculated following the same parameters and software programs as above. We tested for Isolation by distance-related dispersal limitation (IBD) at population level through a Mantel test of pairwise actual genetic (allelic) differentiation using D_est_ [76] (SMOGD online) versus pairwise direct flight geographical distances (km) with 9999 permutations in GenAlex. Considering the LDD potential of *Rhizophora* species and the regional scale of our study (*i*.*e*., 11 study sites located along 400 km of coastline), we decided to check whether or not dispersal is limited by physical barriers rather than by geographic distance. We therefore calculated barriers to gene flow using the software program Barrier v.2.2 [77] based on information from 3 matrices (Fst, D_est_, and Nei’s genetic distances between populations) following an *a priori* assignment of 3 barriers: (1) the CVL; (2) the ocean current convergence zone; and (3) geographical distance. This program runs the Monmonier maximum difference algorithm on a genetic distance matrix (or matrices) and geographical coordinates of sampling locations to detect genetic breaks (*i*.*e*., genetic barriers) based on *a priori* number of barriers set by the user. Due to the likely inbreeding signals observed in the Kribi population, this population was omitted and a second run was conducted using Barrier to assess whether or not the barriers observed in the first run were due to inbreeding signals. Also, to identify the major barrier to gene flow we did a third run with only one *a priori* barrier set. Following Dodd *et al*. [36] and Wee *et al*. [53], and similar to the first hypothesis above, a new predictor matrix of population pairs on either side and on the same side of this convergence zone, was created, which was used in a Mantel test with a pairwise Fst matrix. We also carried out a partial Mantel test of three matrices: genetic distance matrix (Fst), geographic distance matrix, and the predictor matrix in IBDWS v. 3.23 online [78]. This allowed for investigating the significance of this convergence zone of ocean currents as a barrier to gene flow. To ascertain this, genetic differentiation (Fst) for all population pairs from within the same ocean current were classified as “Within” and pairs from different ocean currents (*i*.*e*, from opposite sides of this barrier) as “Between”. Using this classification, the two groups were tested for significant difference in Fst using the Mann–Whitney U test in Statistica v.8 (StatSoft Inc., USA).

Additionally, gene flow patterns and genetic clusters from the STRUCTURE analysis were interpreted in the context of ocean surface currents from a mesoscale and tide-resolving configuration of the Massachusetts Institute of Technology general circulation model (MITgcm; [79]). The model has horizontal grid spacing of *ca*. 4 km and vertical grid spacing of 1 m near the surface in order to better resolve surface boundary layer currents. The simulation is initialized from a data-constrained global ocean solution provided by the Estimating the Circulation and Climate of the Ocean, Phase II (ECCO2) project [80]. At the surface the simulation is forced by six-hourly fields from the 0.14° (*i*.*e*., 15 km or less) European Center for Medium-Range Weather Forecasts (ECMWF) atmospheric operational model analysis, starting in 2011. Tidal forcing for the 16 largest tidal constituents is also applied. The bathymetry is a blend of the Smith and Sandwell [81] version 14.1 and the International Bathymetric Chart of the Arctic Ocean (IBCAO).

Virtual propagules were released hourly for the months February, March, April, September and October 2012, being the general propagule release periods for *R*. *racemosa* in the study area (personal observation; observations in line with Menezes *et al*. [82] for Brazil). In total, 3626 virtual propagules were released in each of the 11 coastal locations. All virtual propagules were given a floating period of 3 months and were advected by surface currents from the numerical ocean simulation described above. These virtual 3-month propagule trajectories are used to interpret gene flow patterns and genetic clusters in this study.

#### The role of ocean current convergence on genetic diversity level

It is expected that at the convergence zone of both the Guinea and northward flowing ocean currents, genetic diversity will be higher because of the input of alleles (via propagules) from northerly and southerly mangrove populations. To test this, we pooled populations into estuary groups and tested for significant differences between groups in allelic richness, genotypic diversity and heterozygosity deficiency (Fis) in FSTAT. Since the Lokoundje and the Ntem Estuaries had just one population each, they were pooled into one group since they lie on the same trajectory of the northward flowing surface current (on the same side of the convergence zone).

## Results

### Data quality and genetic diversity

We found no linkage disequilibrium between any pair of loci. Null alleles could potentially be present in 3 loci at Kribi (site 10, frequency < 0.4) (S2 Table). Significant deviations from HWE were observed in 2 (Rrace5 and Rrace7) out of 11 Loci (p < 0.001), following the multi-population tests. Genetic diversity of *R*. *racemosa* populations from the Cameroonian coastline was low to moderate. The observed heterozygosity of the populations ranged from 0.169 to 0.369 (mean = 0.295) while the expected heterozygosity (He) ranged from 0.244 to 0.335 (mean = 0.290) (Table 2). The average allelic richness was 2.6 and was highest at BERI (site 8, Ar = 3.0), BEK (site 3, Ar = 2.9) and TIKO (site 5, Ar = 2.9). Kribi (site 10) had the lowest allelic richness (Ar = 2.1) and heterozygosity (Ho = 0.169) and was the only population with significant inbreeding coefficient (Fis = 0.316, p <0.05). Five out of 11 populations gave signatures of recent bottlenecks (p < 0.001 under the standardized difference test for SMM) (Table 2).

**Table 2:** Descriptive statistics of genetic diversity based on 11 nuclear microsatellite loci for 11 populations of *Rhizophora racemosa* from the Coast of Cameroon.

Site No.	Pop ID	Ntransect	N	At	Ae	Ar	Ho	He	Fis
**1**	**EKO**	1	30	28	1.6	2.5	0.339	0.312	-0.07
**2**	**MBO**	3	91	32	1.6	2.5	0.364	0.309	-0.17
**3**	**BEK**^b^	4	125	43	1.5	2.9	0.281	0.276	-0.012
**4**	**MAB**	1	31	27	1.4	2.4	0.284	0.25	-0.121
**5**	**TIKO**^b^	5	126	38	1.6	2.9	0.312	0.321	0.031
**6**	**AKN**^b^	3	125	36	1.6	2.7	0.281	0.285	0.02
**7**	**SADI**	5	141	36	1.6	2.7	0.302	0.309	0.024
**8**	**BERI**^b^	4	122	40	1.7	3	0.369	0.335	-0.095
**9**	**RSVM**	3	80	29	1.5	2.4	0.266	0.257	-0.028
**10**	**KRIBI**	2	57	25	1.5	2.1	0.169	0.244	0.316*
**11**	**CAMPO**^b^	2	52	34	1.5	2.8	0.281	0.285	0.027
	Mean	3	89	33.5	1.6	2.6	0.295	0.289	-0.039
** **	SE	0.4	4	1.7	0	0.1	0.022	0.019	0.038

^b^ indicates populations undergoing recent bottleneck (standardized difference test; p < 0.001), while

* indicates populations with significant Fis (p <0.05). N = sample size, Ntransect = number of transects per population, At = total number of alleles, Ae = number of effective alleles, Ar = allelic richness, Ho = observed heterozygosity, He = expected heterozygosity, Fis = inbreeding coefficient.

### Genetic structure

**Fig 3:**
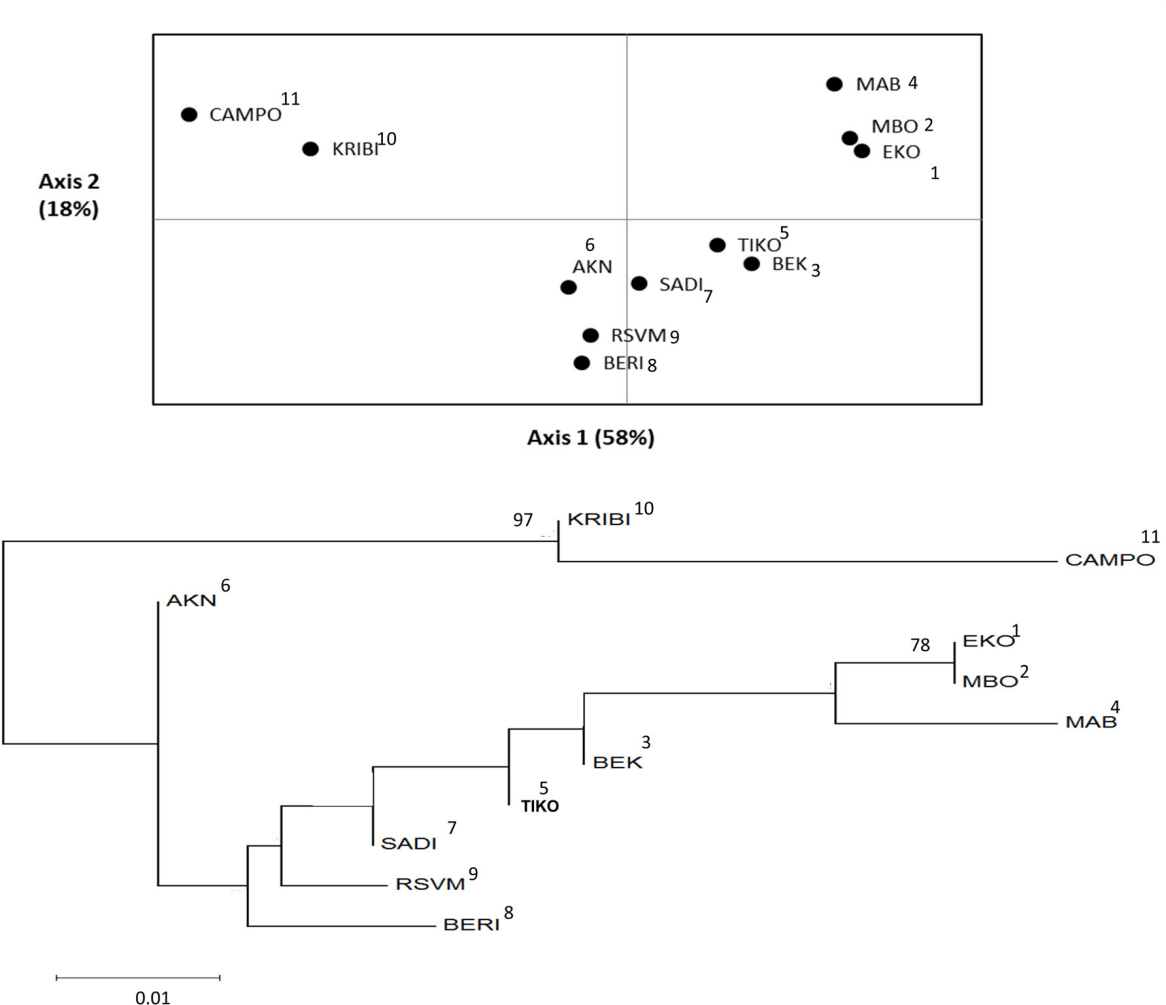
Principal Coordinate analysis (PCoA, above) and Neighbor-joining tree (NJ tree, below). The grouping of 11 populations of *Rhizophora racemosa* along the entire coast of Cameroon into 3 groups with high admixtures of populations from the Rio Del Rey Estuary and the Cameroon Estuary complex and isolation of first group of populations of the Loukondje Estuary (Kribi, site 10) and the Ntem Estuary (Campo, site 11). The second group is made-up of 2 landward populations from the Rio Del Rey Estuary (MBO, site 2 and EKO, site 1) (>75% bootstrapping). A third large group consisting of one seaward population from the Rio Del Rey Estuary (BEK, site3) and 5 other populations from the Cameroon Estuary complex (sites 5–9). Bootstrap values ≥ 75 are indicated on each node of the NJ tree and site numbers (1–11) are indicated beside the pop ID’s.

Jackknifing across loci showed that there was low overall genetic differentiation (Ѳ = 0.073, SE = 0.019) and no inbreeding (f = -0.007, SE = 0.054). However, there was 6% variation among regions and 4% among populations following a hierarchical AMOVA-Fst (Table 3). Frt (0.059) and Fst (0.098) were both low but significant at p < 0.001. The non-hierarchical AMOVA-Fst showed 8% variation among populations (Fst = 0.076) (Table 3). Bayesian clustering analysis assembled individuals into 2 clusters (K = 2, S1 Fig), with a very high degree of admixture of populations from the Rio Del Rey Estuary and those of the Cameroon Estuary complex (CEC) while those of the Lokoundje and the Ntem estuaries formed a different cluster (Fig 2). Pairwise Fst and D_est_ generally increased significantly (p < 0.001) with geographical distance of population pairs (Table 4). PCoA and NJ tree showed a complex spatial relationship among populations. Generally patterns were similar to those observed for Bayesian clustering analysis, as there was clustering (*i*.*e*., mixtures) of populations of the Rio Del Rey Estuary and those of the CEC while the Kribi and Campo (sites 10 and 11) populations of the Lokoundje and the Ntem estuaries respectively were grouped separately (Fig 3).

**Table 3:** Analysis of Molecular Variance (AMOVA-Fst) based on a pooling of populations (Hierarchical) into estuaries and non-pooling (non-hierarchical).

Hierarchical based on grouping into estuaries						Non-hierarchical					
Source	df	SS	MS	Est. Var.	%	Source	df	SS	MS	Est. Var.	%
**Among Estuaries**	3	149.164	49.721	0.109	6%	**Among Pops**	10	254.577	25.458	0.136	8%
**Among Pops**	7	105.412	15.059	0.072	4%	**Among Indiv**	971	1620.773	1.669	0.008	0%
**Among Indiv**	971	1620.773	1.669	0.008	0%	**Within Indiv**	982	1622.5	1.652	1.652	92%
**Within Indiv**	982	1622.5	1.652	1.652	90%	**Total**	1963	3497.849		1.797	100%
**Total**	1963	3497.849		1.842	100%	**F-Statistics**	**Value**	**P(rand > = data)**			
**F-Statistics**	**Value**	**P(rand > = data)**				**Fst**	0.076	0.000			
**Frt**	0.059	0.001				**Fis**	0.005	0.293			
**Fsr**	0.041	0.001				**Fit**	0.08	0.000			
**Fst**	0.098	0.001				**Nm**	3.0				
**Fis**	0.005	0.304				**F'st**	0.108				
**Fit**	0.103	0.001									
**Nm**	2.3										

**Table 4:** Pairwise population genetic differentiation.

	EKO	MBO	BEK	MAB	TIK	AKN	SADI	BERI	RSVM	KRIBI	CAMPO
**EKO**	0	0	0.001	0.006	0.003	0.01	0.004	0.014	0.015	0.055	0.062
**MBO**	0.000^*ns*^	0	0.002	0.009	0.003	0.01	0.006	0.017	0.018	0.052	0.063
**BEK**	0.040	0.044	0	0.013	0.003	0.004	0.003	0.009	0.009	0.035	0.044
**MAB**	0.054	0.056	0.087	0	0.013	0.018	0.017	0.032	0.015	0.038	0.051
**TIK**	0.028	0.029	0.019	0.060	0	0.004	0.003	0.005	0.008	0.03	0.046
**AKN**	0.087	0.085	0.036	0.114	0.021	0	0.001	0.001	0.005	0.015	0.029
**SADI**	0.064	0.068	0.014	0.101	0.017	0.015	0	0.003	0.004	0.027	0.039
**BERI**	0.094	0.099	0.062	0.134	0.040	0.028	0.042	0	0.007	0.028	0.039
**RSVM**	0.110	0.118	0.078	0.122	0.048	0.044	0.057	0.044	0	0.021	0.024
**KRIBI**	0.202	0.190	0.164	0.204	0.121	0.086	0.115	0.112	0.121	0	0.009
**CAMPO**	0.239	0.234	0.217	0.265	0.177	0.133	0.155	0.153	0.176	0.062	0

(Fst) (below the diagonal) and allelic differentiation (D_est_) (above the diagonal) between all possible pairs of populations. All significant at p < 0.001 and ^*ns*^ means non-significant differentiation between MBO and EKO

### Hypotheses 1 and 2: Corridors for and barriers to gene flow

**Fig 4:**
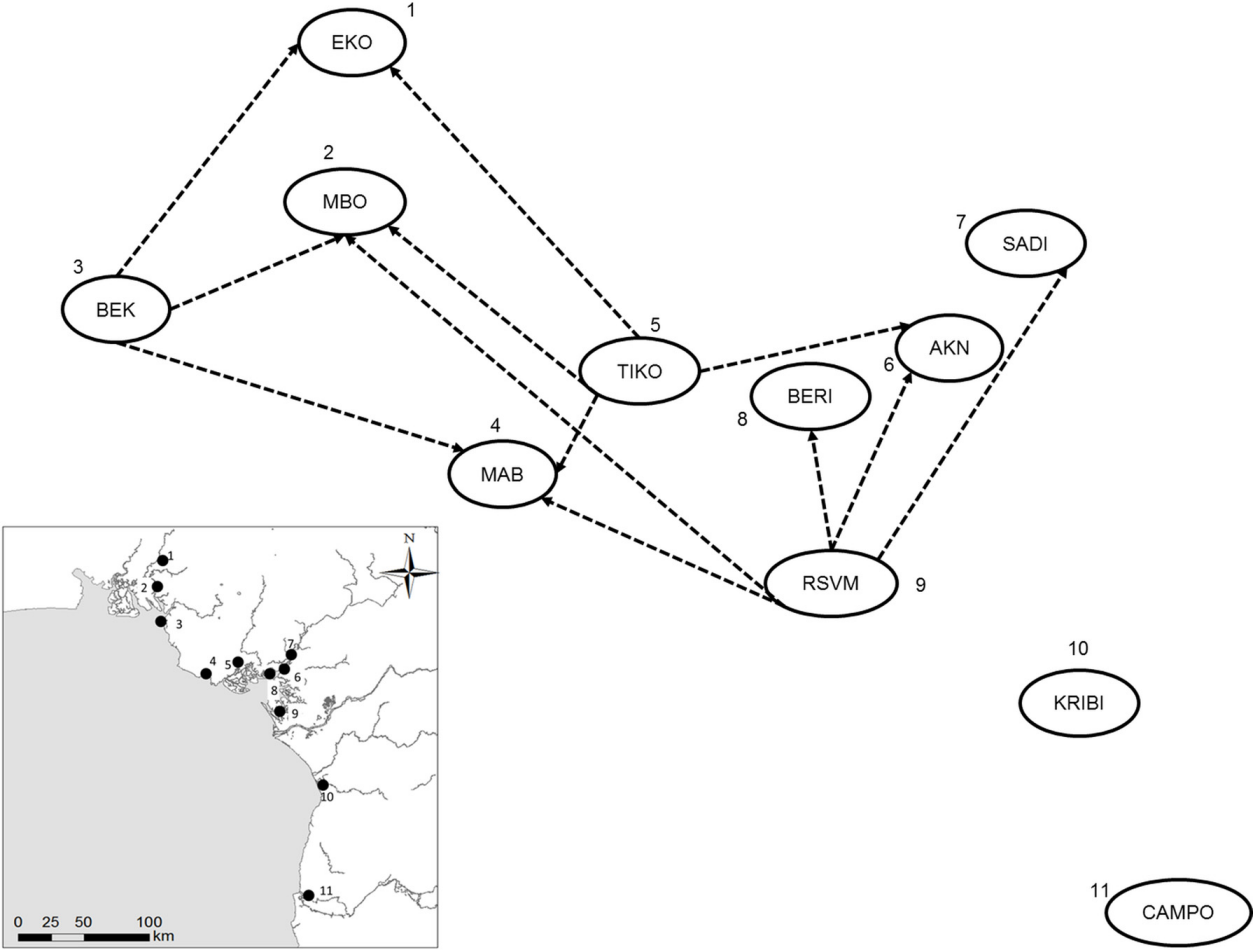
Pairwise contemporary migration rates between populations based on Bayesian estimates using individual multilocus genotypes.

**Fig 5:**
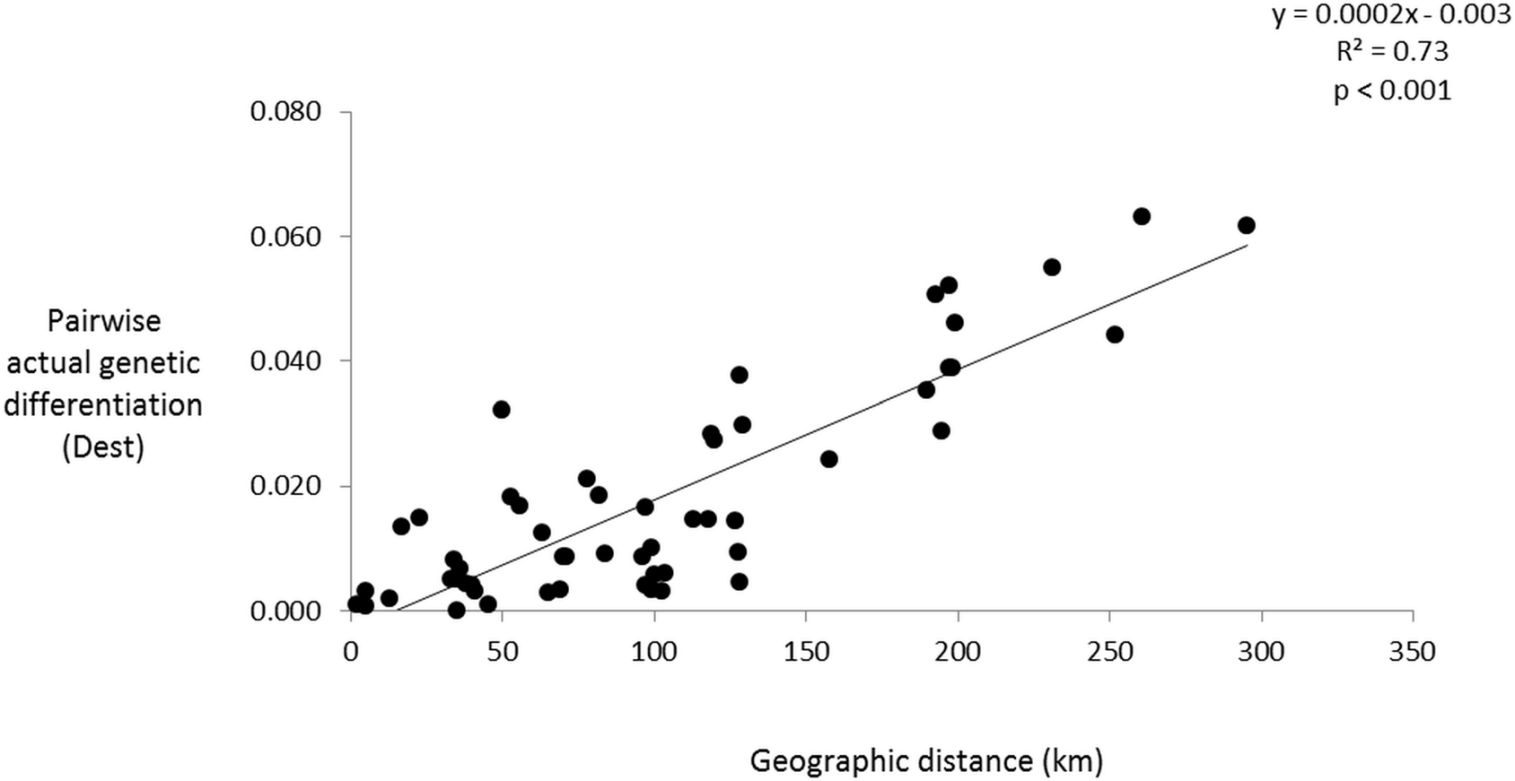
Strong isolation by distance (IBD) among *Rhizophora racemosa* populations along the coast of Cameroon. The pairwise actual genetic differentiation (D_est_) increases from 0 to 7% over a direct flight geographic distance of up to 300 km), p < 0.001.

**Fig 6:**
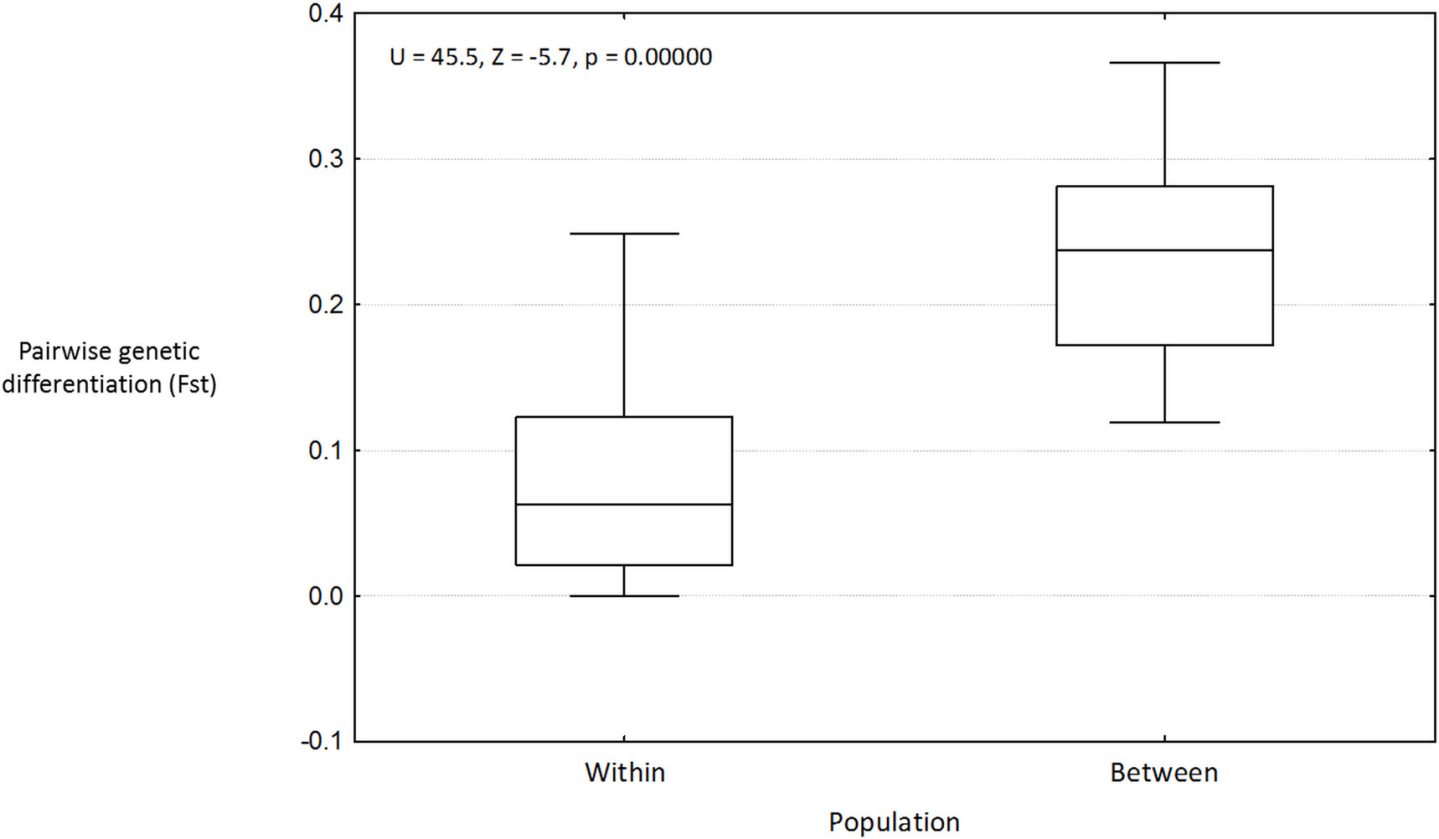
Box-whisker plot of mean population pairwise Fst values between “Within” ocean current population pairs group and ‘Between’ ocean current population pairs group.

**Fig 7:**
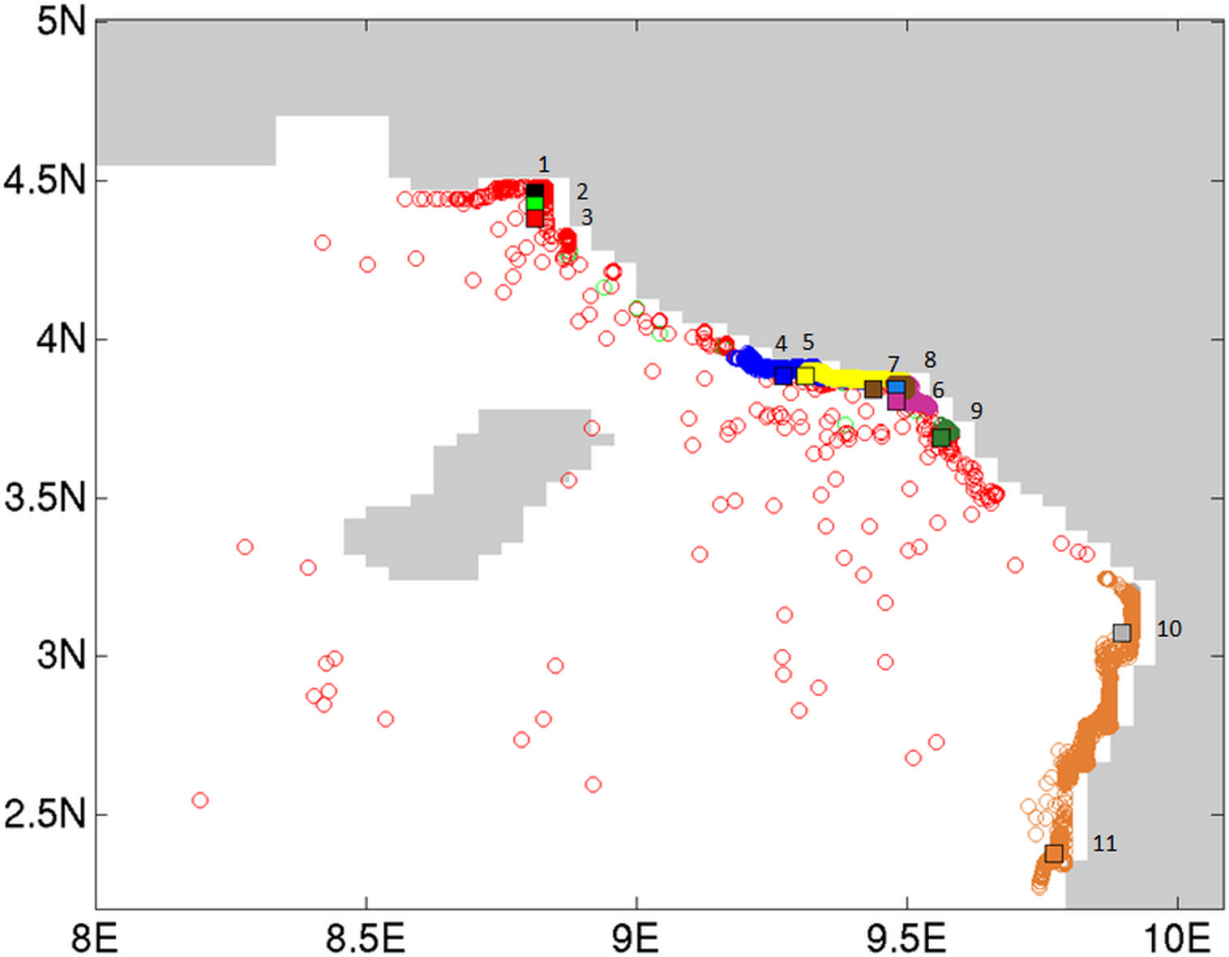
Location of virtual propagules (dots) after 3 months of floating. Virtual propagules were released hourly during the months February, March, April, September and October 2012, since these months correspond with propagule release periods for *Rhizophora racemosa* in the study area (personal observation). Hence, in total, 3626 virtual propagules were released in each of the locations. Author-defined release locations correspond with the localities where samples for our genetic analyses were collected, which were subsequently shifted to the closest ocean point (rectangles) to ensure the possibility of particle movement. Site numbers (1–11) are indicated beside the corresponding point.

**Fig 8:**
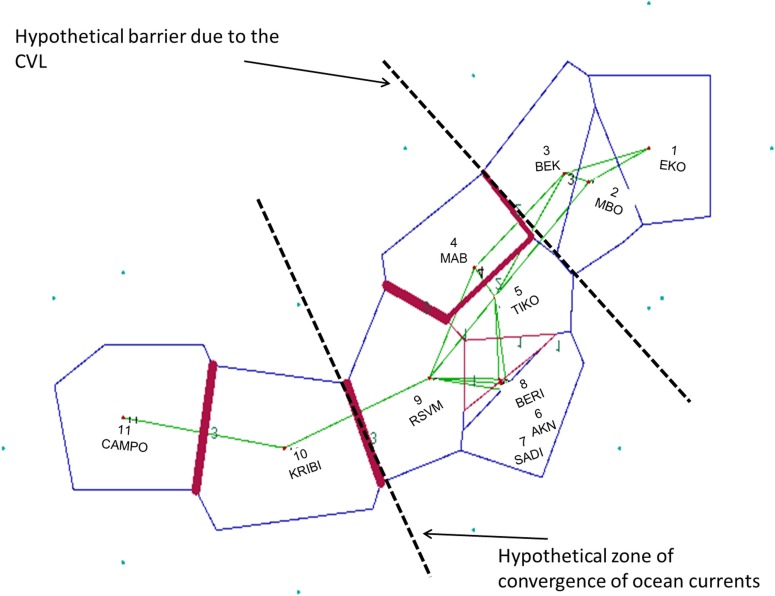
Gene flow barriers (red lines) and hypothetical barriers (Black dotted lines). The thickness of the red line indicates the importance of the barriers based on the 3 different distance matrices (pairwise Nei’s genetic distances between populations (S3 Table), pairwise Fst, Pairwise D_est_) used in the analysis.

A Mantel test of the predictor matrix assuming the Cameroon Volcanic line (CVL) is a barrier to gene flow and pairwise Fst was non-significant (data not shown). Contemporary migration rates (Fig 4, S1 Table) were high between populations of the Rio Del Rey and CEC, while those of the Lokoundje and the Ntem estuaries are isolated from the others (no migration). These results are in line with the genetic structure pattern, where high levels of admixture among populations of the Rio Del Rey and CEC were observed. The IBD test was significant (Fig 5, S3 Fig), indicating dispersal limitation with geographical distance. Assuming the convergence of ocean currents around the CEC to be a barrier to gene flow, the predictor matrix following this barrier and pairwise Fst in a Mantel test was significant (R^2^ = 0.631, p < 0.05). Partial mantel test was significant for genetic and geographic distances while controlling for the predictor matrix (r = 0.53, p < 0.05), as well as for genetic distance and predictor matrices, while controlling for geographical distance (r = 0.43, p < 0.05). This significance was confirmed by a significant Mann–Whitney U test (p < 0.001) when Fst of population pairs were grouped into pairs from within the same ocean currents (“Within”) and pairs from across the convergence zone barrier (“Between”) (Fig 6). Probabilistic estimates of propagule dispersal trajectories from our model suggest that propagules can go through the Inter-Bioko-Cameroon (IBC) corridor; they also suggest that, Bekumu (site 3) and, secondarily, Mbongo (site 2) are potentially major source populations of propagules from the Rio Del Rey to the CEC (Fig 7). Model results also show that the convergence zone of currents represents a barrier to gene flow, as propagules from either side do not cross this barrier (Fig 7). Propagules from the CEC cover the shortest dispersal distances (Fig 7). A more holistic test to identify barriers to gene flow showed that the convergence zone of ocean currents is a barrier to gene flow (Fig 8), even after omitting the only (potentially) inbred population (Kribi, site 10) and after only one *a priori* barrier was used in the analyses (data not shown). Additionally, this test did not identify the CVL as a barrier to gene flow (Fig 8). Mabeta (site 4) was also isolated from all other populations (Fig 8). Ocean current patterns also illustrate the established IBC corridor is a pathway for connectivity between the CEC and the Rio Del Rey Estuary, and indicates as well the zone of convergence of the two ocean currents (S2 Fig).

### Hypothesis 3: Increased genetic diversity by convergence of two ocean currents at the Cameroon Estuary complex

Despite the large sample size of the Cameroon Estuary complex (CEC), there was no significant difference in genetic diversity parameters (Ar, Ho, Hs) among estuaries (Table 5). This indicates that there is no accumulation of genetic diversity in the CEC due to the convergence of ocean currents around this area (Table 5) or any other process. However, there was significant difference in inbreeding coefficient among estuary groups (Fis, at p < 0.005).

**Table 5:** Descriptive statistics of comparative genetic diversity of different estuaries.

Estuary	N	Ar	Ho	Hs	Fis*	Fst	Rel
**Rio Del Rey**	246	2.8	0.319	0.295	-0.082	0.035	0.074
**CEC**	627	2.7	0.307	0.304	-0.013	0.04	0.079
**Ntem & Lokoundje**	109	2.5	0.222	0.267	0.167	0.065	0.106

* Significance at p < 0.01 (p = 0.004).

## Discussion

### The Inter-Bioko-Cameroon corridor facilitates gene flow between mangroves of the Rio Del Rey and the Cameroon Estuary complex

Our results are in line with the hypothesis that contemporary global mangrove distribution is shaped by (historical) vicariance and LDD [24, 39], and suggests that this applies as well to regional and local scales [62]. Bayesian clustering analysis shows high admixture of populations of the Rio Del Rey Estuary and those of the Cameroon Estuary complex (CEC). This is in accordance with the observed patterns of contemporary migration rates and modeled propagule dispersal trajectories and suggests that post-glacial SLR has established the genetic connectivity between populations of the Rio Del Rey Estuary and those of the CEC via the Inter-Bioko-Cameroon (IBC) corridor. *Rhizophora racemosa* is wind pollinated, with occasional insect pollination [83, 84]. Pollen dispersal by wind covers certain distances, but unlikely over the entire studied area. The efficiency of wind pollination is low in fragmented landscapes. This would imply restricted pollen dispersal for wind pollinated *Rhizophora* species, since mangroves are naturally patchy [85]. Additionally, mangrove habitats are generally heterogeneous landscapes, (*e*.*g*. interspersed by the landscape matrix of other forest or vegetation types) and this presents another strong barrier for most insect pollinators [85]. Furthermore, the (historical) landscape was mountainous in nature (see Cameroon Volcanic Line). Although palaeo-weather conditions and wind directions are not known, this mountainous terrain (by acting as a wind shield) would have hardly allowed wind-borne pollen to cross over between the two formally separated estuaries. Nevertheless, potential gene flow between these two estuaries prior to the LGM cannot be completely excluded. Occasional fluctuations in sea levels prior to the LGM could have allowed for propagule exchange via the IBC corridor before its complete formation 12 ka BP. However, a period of 12 ka (*i*.*e*., onset of the formation of the IBC corridor) provides a sufficiently long time period to generate the observed patterns of high connectivity between the Rio Del Rey and the CEC. This has resulted in reduced genetic differentiation between population pairs of the CEC and the Rio Del Rey, indicating that dispersal of propagules is more effective than pollen at preventing genetic divergence of populations (promoting homogeneity) [86]. Since seeds carry two alleles, their dispersal is twice as effective as the gene flow by pollen dispersal (if selection is ignored) [86]. Post-glacial mangrove shifts and expansion have mostly been attributed to the formation of favorable habitats by warmer temperatures at higher latitudes [8–10, 25, 27–29], accretion and favorable topographical conditions at landward margins [1, 7, 87]. Here, we present a case of connectivity between two mangrove estuaries through the formation of a corridor for gene flow, following post-glacial SLR. Our results from the Mantel tests, pairwise Fst, as well as the test of genetic barriers, indicate that the Cameroon Volcanic Line (CVL) does not represent a barrier to gene flow between these two mangrove areas. Historically (> 13 ka BP), however, connecting Bioko Island to mainland Cameroon, the CVL presented an important land barrier that isolated the Rio Del Rey mangroves from those situated south of this barrier, since regional uplift and resulting land barriers are among the few impenetrable barriers to gene flow among seafaring organisms [50]. The present-day absence of this barrier underscores the functional role of the recently (< 12 ka BP) formed corridor following SLR, in facilitating gene flow between these two estuaries. The land bridge has not left any trace in genetic differentiation of mangrove populations of these two estuaries. Our results also show that (virtual) propagules of the CEC presently cover the shortest dispersal distances. This may be due to the tidal regimes in this area, preventing propagules from reaching the open ocean [62], hence reducing their potential to embark on LDD. The role of tides in facilitating bidirectional gene flow along the Wouri River channel, for example, was highlighted by Ngeve *et al*. [62]. Understanding plant dispersal in riparian habitats provides important knowledge to understand how these areas are and had been colonized, and provides a hint on how they may respond to climate change [88]. Contemporary migration rates indicate that colonization of the Wouri River channel may have occurred following Slatkin’s “propagule pool” model [42]) with the RSVM (site 9) (mangroves of the Douala-Edea Reserve) population being the most likely propagule source. Other populations of the CEC and the Rio Del Rey Estuary may have followed the “migrant pool” model [42]. Nevertheless, our results do not allow for conclusions on the colonization model for the southernmost and more diverged Kribi (site 10) and Campo (site 11) populations. Their source populations were not revealed, but it is likely that they have originated from mangroves in the Angola-Congo-Gabon range, borne by the northwards flowing surface currents.

Despite the resilience demonstrated by mangrove ecosystems to climate change, Ellison and Zouh [89] showed that recent climate change-induced SLR in Cameroon has resulted in diebacks in over two-third of seaward mangroves at a rate of about 3 m shoreline per year over the last 3 decades. According to Alongi [2] mangroves around the Cameroonian range (Central Africa) are among the least vulnerable to the effects of climate change globally, although reduction in subsidence, saline intrusion and SLR are assumed to be the most likely threats to negatively impact these mangroves [5]. However, we have indications that lowlands hampering gene flow during glacial times have been submerged by SLR, resulting in the connectivity of distantly located estuaries. Seasonal floods, expected to increase with changing climate, enhance the accessibility of mangrove forest by inhabitants of local communities, hence promoting mangrove exploitation [90]. Therefore, climate change, in addition to its impact on the area, on spatial distribution of suitable mangrove habitat and dispersal barriers, may influence the impacts from other anthropogenic drivers [2]. Future biogeographical range shifts will depend as well on the spatial variability of local extinction risk in response to environmental changes [5, 24], and other factors such as the geomorphological features of the locality [5].

The consequences of the IBC corridor for local mangroves are not obvious, since gene flow can either limit evolution by preventing local adaptation or facilitate evolution by spreading new genes and gene combinations throughout a range [91], except in areas where environmental constraints exert a selection pressure. However, the isolation of Bioko Island may be a remnant of range fragmentation of mangroves, at least if we assume that mangrove forests were ubiquitous in this region during glacial times. This is for example the case for the insect *Glossina palpalis palpalis*, where Bioko populations have been isolated, resulting in a unique clade, different from mainland populations [58]. Therefore, future studies should consider mangrove samples from Bioko to assess the full impact of the IBC corridor on genetic connectivity and potential range fragmentation of Central African mangrove populations. Nevertheless, there are indications that LDD via the IBC corridor, in addition to the time of its existence (*ca*. 12 ka), override patterns that might have existed prior to the LGM.

### Ocean current patterns as a determinant of genetic structure

As hypothesized, ocean currents constitute an important determinant in understanding the observed regional genetic structure. This is expected, since ocean currents, in addition to pollen flow, represent the primary mechanism via which genetic material is exchanged among populations, close and remote. Ocean current patterns in our study area seem to underscore the genetic clusters that were detected using the Bayesian clustering, the pairwise Fst and D_est_. Both the output from tests with genetic data and our probabilistic estimates from the numerical dispersal model, for example, support the movement of propagules from Bekumu (site 3) to the more southerly (*ca*. 100 km) situated CEC. The southernmost populations, Kribi (site 10) and Campo (site 11), on the other hand seem to be lying “oceanographically” beyond the reach of the other populations in this region, explaining their genetic isolation.

Interestingly, while our genetic results reflect the absence of a contemporary barrier between the Rio Del Rey and CEC, numerical model output indicates that the genetic discontinuity between the CEC and the Kribi (site 10) and Campo (site 11) populations aligns with present-day ocean circulation in this area. Here, a linear convergence zone results from the confluence of ocean currents from the north and south. Ocean currents at the frontal zone are directed seaward, consequently preventing the exchange of propagules between localities north and south of it. The importance of considering ocean current patterns in explaining the genetic structure of coastal ecosystems has also been postulated by Wee *et al*. [53], who demonstrated that the genetic discontinuity between the mangrove populations of the Andaman Sea and the Malacca Strait is maintained by ocean features that prevent the mixing of water between these areas.

Considering ocean current velocities of 0.1 to 0.2 m s^-1^ and geographical distances among the various estuaries in our study area (Rio Del Rey, CEC, Lokoundje and Ntem) on the order of 100 km, propagules could be transported between two neighboring populations in about 6 to 12 days. This is indeed an oversimplification, since dispersal vector properties (in this case ocean surface current speed and direction) vary over the course of a propagule’s dispersal trajectory. However, it demonstrates that within reported floating and viability periods for *Rhizophora* species, ranging from several weeks to several months [92–96], there is a sufficiently long period of time to reach remote areas; although our results demonstrate that this does not guarantee connectivity. Hence, the concept of isolation by oceanographic distance (*sensu* Wood [97]), more than just distance, may be a more appropriate and realistic model to explain the genetic structure of seafaring organisms. Since this model takes into account variations in dispersal vector properties, genetic breaks among proximate populations of species with propagules that can float for several months can be much better understood (see also [53]). The importance of oceanographic features, such as cyclonic and anticyclonic eddies in quantifying the dispersal trajectories of biological material at the ocean surface, has been mentioned in earlier oceanographic studies in the Mozambique Channel [98, 99]. This was reported as well by Wee *et al*. [53] in explaining the observed genetic structure in Southeast Asian mangroves. Here, we demonstrate that ocean convergence zones in coastal areas can act as a barrier for connectivity, and underscore the strength and complementarity of genetic and modeling studies. Hence, our study clearly indicates that knowledge of ocean circulation constitutes primary knowledge in the study of observed genetic structure of ocean-dispersed organisms. The combination of genetic data and model output, as well as empirical data from release-recapture methods, has been proposed earlier as a way to gain insight on (long distance) dispersal (see [12, 53, 100]). Others have demonstrated the value and strength of mathematical dispersal models to study oceanographic dispersal and connectivity in coastal areas at regional and global scales [97, 101–104] at which the use of release-recapture methods can hardly be applied (but see [105]).

### Ocean convergence does not increase genetic diversity at the Cameroon Estuary complex

Moderate genetic diversity was observed in the red mangroves of the Cameroonian coastline and there was a general trend of genetic diversity parameters, decreasing from north to south. This may indicate that the relatively smaller populations in the Lokoundje (Kribi site 10) and Ntem (Campo, site 11) estuaries, located on the south of this coastline, have lower effective population sizes. Genetic diversity is directly proportional to effective population size [106, 107]. Several populations were observed to be under recent bottleneck, likely as a result of high anthropogenic pressures on these coastal ecosystems [17, 18, 60, 108, 109]. Heterozygosity was moderate to low, and this has been commonly observed in *Rhizophora* species [62, 110, 111]. Generally, low heterozygosity can be attributed to several factors [*cf*. 36, 47, 48, 110]. Ng *et al*. [110] and Wee *et al*. [111] observed low heterozygosity to be in agreement with the biology of *Rhizophora* species. Null alleles are relatively common in microsatellite analysis [112] and they could be present at some loci in our study populations, albeit at very low frequencies (S2 Table). The number of effective alleles and mean number of alleles in all populations was low (means: Ae = 1.6, Ad = 3) so it is unlikely that null alleles will account for reduced heterozygosity. Null allele frequencies were highest in Kribi (site 10) for the Loci Rrace5 and Rrace6. Rrace5 deviates from HWE and this population has a significant inbreeding coefficient (Fis). So the potentially (relatively) high null allele frequencies at this population are most likely an over estimation because the significant Fis of this population and the deviation of this loci from HWE [113]. Significant Fis in the Kribi (site 10) population could be explained from either Wahlund-effect or potential inbreeding in the small, highly fragmented adult stands that made up the transect from Londji.

The highest genetic diversity was observed in the BERI (site 8), BEK (site 3), and TIK (site 5) populations and this could be due to propagule supply from upstream populations into these seaward populations (BERI and BEK) [62]. These two sites are part of the (CEC). We expected that due to the presence of an oceanographic convergence just off the CEC, genetic diversity would be significantly higher at the CEC via the supply of propagules from both sides. There was no significant difference in the genetic diversity (Ar and Ho) of the CEC over other estuaries, despite the large sample size of the CEC. Thus, locally, river and coastal currents increase genetic diversity of the downstream populations of the Wouri River [62], but regionally, the CEC is not significantly different in genetic diversity from the other studied areas. *Rhizophora* propagules have quicker establishment chances and longer viability periods [114]. Despite the high fecundity of mangroves [115], low seed dispersal effectiveness (SDE) can result when the probability for dispersed propagules to remain viable along their dispersal trajectory is low and the potential for successful establishment after stranding is limited [116]. Hence, a plausible explanation is that many viable propagules are transported to the open sea and sink rather than projected suitable habitats where they could strand and eventually establish.

## Conclusion

We found sufficient evidence to conclude that climate change-induced SLR in the recent past has created a corridor for gene flow between *Rhizophora racemosa* populations of the Rio Del Rey Estuary and those of the Cameroon Estuary complex. In contrast, our study illustrates how ocean features such as convergence zones in coastal areas can greatly influence the overall genetic structure in a particular region. The isolation by distance signals observed is likely isolation by resistance posed by the presence of an oceanic convergence zone, which was found to be an effective barrier to gene flow, hampering the exchange of propagules between populations at both sides of this barrier. Contrary to expectation, the convergence of two ocean currents did not result in higher genetic diversity at the Cameroon Estuary complex, likely as a result of ineffective overall dispersal.

## Supporting Information

S1 Fig Selecting the best K value using the Evanno method of delta K (a) and Ln(P) (b).
(TIF)

S2 Fig Ocean current simulated patterns of the coast of Cameroon. The convergence zone of the two currents offshore of the Cameroon Estuary complex is clearly revealed.
(TIF)

S3 Fig Isolation by Distance based on pairwise Nei’s genetic distances of populations and geographic distance. (TIF)

S1 Table Pairwise contemporary migration rates between population based on Bayesian estimates using individual multilocus genotypes. Significant migration rates are highlighted in bold. **(DOC)**
(DOCX)

S2 Table Estimate of null allele frequency in all Loci for all studied populations. (DOC)
(DOCX)

S3 Table Pairwise Nei’s genetic distances of populations. (DOC)
(DOCX)
