# Supplementary figures and images for: Contrasting Effects of Historical Sea Level Rise and Contemporary Ocean Currents on Regional Gene Flow of Rhizophora racemosa in Eastern Atlantic Mangroves
Source: PLoS One. 2016 Mar 10;11(3):e0150950. doi: 10.1371/journal.pone.0150950 (PMC4786296; doi:10.1371/journal.pone.0150950)

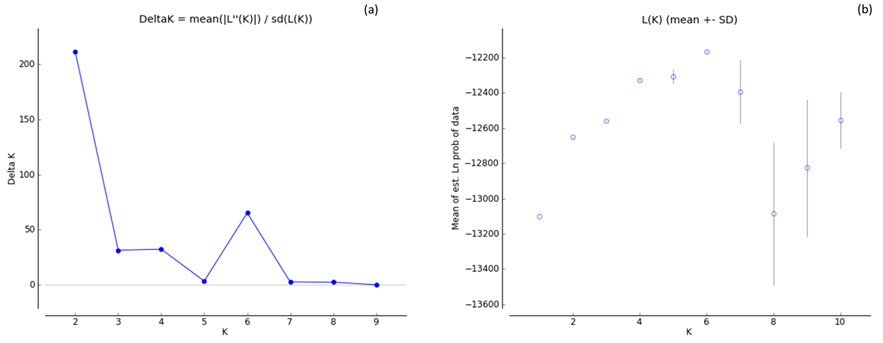

Supplement: S1 Fig — Selecting the best K value using the Evanno method of delta K (a) and Ln(P) (b). (TIF) [file pone.0150950.s001.tif]

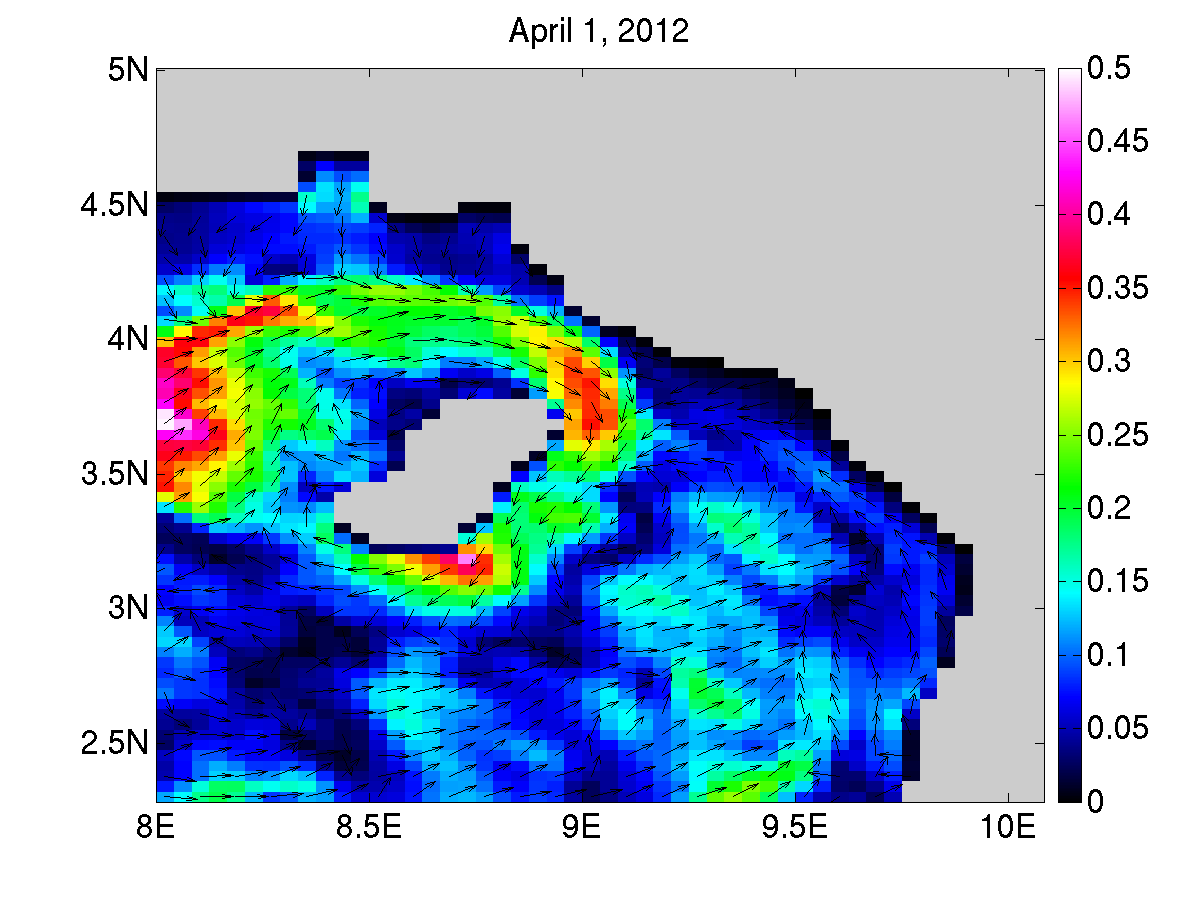

Supplement: S2 Fig — The convergence zone of the two currents offshore of the Cameroon Estuary complex is clearly revealed. (TIF) [file pone.0150950.s002.tif]

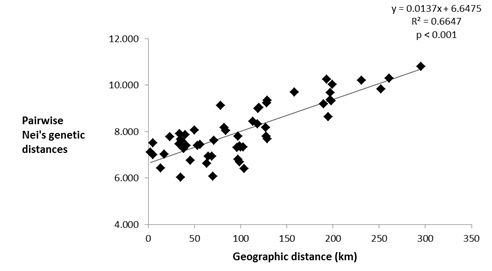

Supplement: S3 Fig — (TIF) [file pone.0150950.s003.tif]
