# Supplementary material for: Contrasting Effects of Historical Sea Level Rise and Contemporary Ocean Currents on Regional Gene Flow of Rhizophora racemosa in Eastern Atlantic Mangroves
Source: PLoS One. 2016 Mar 10;11(3):e0150950. doi: 10.1371/journal.pone.0150950 (PMC4786296; doi:10.1371/journal.pone.0150950)
Supplement: S1 Table — Significant migration rates are highlighted in bold. (DOC) (DOCX) [file pone.0150950.s004.docx]

S1 Table. Pairwise contemporary migration rates between population based on Bayesian estimates using individual multilocus genotypes. Significant migration rates are highlighted in bold.

| From 🡪 | EKO | MBO | BEK | MAB | TIKO | AKN | SADI | BERI | RSVM | KRIBI | CAMPO |
| --- | --- | --- | --- | --- | --- | --- | --- | --- | --- | --- | --- |
| EKO | 0.677 | 0.006 | **0.232** | 0.004 | **0.056** | 0.005 | 0.004 | 0.004 | 0.004 | 0.004 | 0.004 |
| MBO | 0.001 | 0.671 | **0.233** | 0.002 | **0.052** | 0.004 | 0.005 | 0.002 | **0.022** | 0.004 | 0.004 |
| BEK | 0.005 | 0.001 | 0.966 | 0.001 | 0.008 | 0.006 | 0.003 | 0.001 | 0.004 | 0.002 | 0.001 |
| MAB | 0.004 | 0.004 | **0.055** | 0.683 | **0.021** | 0.011 | 0.008 | 0.004 | **0.198** | 0.008 | 0.005 |
| TIKO | 0.001 | 0.001 | 0.002 | 0.001 | 0.989 | 0.001 | 0.001 | 0.001 | 0.003 | 0.001 | 0.001 |
| AKN | 0.001 | 0.001 | 0.003 | 0.001 | 0.024 | 0.679 | 0.001 | 0.004 | **0.281** | 0.002 | 0.002 |
| SADI | 0.002 | 0.001 | 0.003 | 0.001 | 0.005 | 0.009 | 0.675 | 0.001 | **0.301** | 0.002 | 0.002 |
| BERI | 0.001 | 0.001 | 0.005 | 0.001 | 0.012 | 0.003 | 0.003 | 0.677 | **0.290** | 0.002 | 0.006 |
| RSVM | 0.001 | 0.000 | 0.001 | 0.000 | 0.001 | 0.001 | 0.001 | 0.001 | 0.995 | 0.001 | 0.001 |
| KRIBI | 0.001 | 0.001 | 0.001 | 0.001 | 0.001 | 0.001 | 0.001 | 0.001 | 0.001 | 0.989 | 0.002 |
| CAMPO | 0.001 | 0.001 | 0.001 | 0.001 | 0.001 | 0.002 | 0.001 | 0.001 | 0.003 | 0.002 | 0.987 |
