# Supplementary material for: Contrasting Effects of Historical Sea Level Rise and Contemporary Ocean Currents on Regional Gene Flow of Rhizophora racemosa in Eastern Atlantic Mangroves
Source: PLoS One. 2016 Mar 10;11(3):e0150950. doi: 10.1371/journal.pone.0150950 (PMC4786296; doi:10.1371/journal.pone.0150950)
Supplement: S2 Table — (DOC) (DOCX) [file pone.0150950.s005.docx]

S2 Table. Estimate of null allele frequency in all Loci for all studied populations

| Locus | EKO | MBO | BEK | MAB | TIK | AKN | SADI | BERI | RSVM | KRIBI | CAMPO |
| --- | --- | --- | --- | --- | --- | --- | --- | --- | --- | --- | --- |
| Rrace_1 | 0 | 0 | 0 | 0 | 0 | na | na | na | na | na | na |
| Rrace_3 | 0.0266 | 0.0092 | 0.0339 | 0.0812 | 0.0525 | 0 | 0 | 0 | 0.0002 | 0.1375 | 0 |
| Rrace_5 | 0.1272 | 0 | 0.0787 | 0.0093 | 0.0905 | 0.0527 | 0.0785 | 0.0806 | 0.0651 | 0.4729 | 0.1794 |
| Rrace_6 | 0 | 0.0394 | 0.0602 | 0.068 | 0.0104 | 0.1529 | 0.0989 | 0 | 0 | 0.3602 | 0.2593 |
| Rrace_7 | 0.0956 | 0 | 0 | na | 0 | 0.0468 | 0 | 0.0162 | na | 0 | 0 |
| Rrace_12 | 0.0749 | 0 | 0 | 0.0184 | 0 | 0 | 0 | 0.0265 | 0 | 0 | 0 |
| Rrace_15 | 0 | 0 | 0 | 0.0992 | 0 | 0 | 0.0473 | 0 | 0 | 0 | 0.0272 |
| Rrace_17 | 0.0636 | 0 | 0.0267 | 0.0678 | 0 | 0 | 0.0373 | 0 | 0 | 0.0163 | 0 |
| Rrace_18 | 0 | 0 | 0.0371 | 0 | 0 | 0 | 0 | 0.0531 | 0.0914 | 0.0416 | 0 |
| Rrace_20 | 0 | na | 0 | na | na | na | na | 0 | na | na | 0.0951 |
| Rrace_24 | 0 | 0 | 0 | 0 | 0.1153 | 0 | 0 | 0 | 0.0081 | 0.1342 | 0 |
