# Supplementary material for: Contrasting Effects of Historical Sea Level Rise and Contemporary Ocean Currents on Regional Gene Flow of Rhizophora racemosa in Eastern Atlantic Mangroves
Source: PLoS One. 2016 Mar 10;11(3):e0150950. doi: 10.1371/journal.pone.0150950 (PMC4786296; doi:10.1371/journal.pone.0150950)
Supplement: S3 Table — (DOC) (DOCX) [file pone.0150950.s006.docx]

|  | EKO | MBO | BEK | MAB | TIKO | AKN | SADI | BERI | RSVM | KRIBI | CAMPO |
| --- | --- | --- | --- | --- | --- | --- | --- | --- | --- | --- | --- |
| EKO | 0.000 |  |  |  |  |  |  |  |  |  |  |
| MBO | 6.021 | 0.000 |  |  |  |  |  |  |  |  |  |
| BEK | 6.770 | 6.434 | 0.000 |  |  |  |  |  |  |  |  |
| MAB | 6.406 | 6.065 | 6.625 | 0.000 |  |  |  |  |  |  |  |
| TIKO | 7.330 | 6.927 | 6.934 | 7.016 | 0.000 |  |  |  |  |  |  |
| AKN | 7.799 | 7.370 | 6.796 | 7.392 | 7.271 | 0.000 |  |  |  |  |  |
| SADI | 7.688 | 7.333 | 6.697 | 7.449 | 7.403 | 6.999 | 0.000 |  |  |  |  |
| BERI | 8.174 | 7.806 | 7.304 | 8.059 | 7.673 | 7.102 | 7.512 | 0.000 |  |  |  |
| RSVM | 8.446 | 8.175 | 7.630 | 7.770 | 7.913 | 7.464 | 7.858 | 7.601 | 0.000 |  |  |
| KRIBI | 10.215 | 9.686 | 9.196 | 9.233 | 9.347 | 8.323 | 9.030 | 8.985 | 9.117 | 0.000 |  |
| CAMPO | 10.792 | 10.288 | 9.823 | 10.245 | 10.022 | 8.645 | 9.328 | 9.358 | 9.691 | 8.030 | 0.000 |

S3 Table. Pairwise Nei’s genetic distances of populations
